# How Pig Sperm Prepares to Fertilize: Stable Acrosome Docking to the Plasma Membrane

**DOI:** 10.1371/journal.pone.0011204

**Published:** 2010-06-18

**Authors:** Pei-Shiue Tsai, Núria Garcia-Gil, Theo van Haeften, Bart M. Gadella

**Affiliations:** 1 Department of Biochemistry and Cell Biology, Graduate School of Animal Health, Faculty of Veterinary Medicine, Utrecht University, Utrecht, The Netherlands; 2 Department of Farm Animal Health, Graduate School of Animal Health, Faculty of Veterinary Medicine, Utrecht University, Utrecht, The Netherlands; Institut Européen de Chimie et Biologie, France

## Abstract

**Background:**

Mammalian sperms are activated in the oviduct. This process, which involves extensive sperm surface remodelling, is required for fertilization and can be mimicked under *in vitro* fertilization conditions (IVF).

**Methodology/Principal Findings:**

Here we demonstrate that such treatments caused stable docking and priming of the acrosome membrane to the apical sperm head surface without the emergence of exocytotic membrane fusion. The interacting membranes could be isolated as bilamellar membrane structures after cell disruption. These membrane structures as well as whole capacitated sperm contained stable ternary *trans*-SNARE complexes that were composed of VAMP 3 and syntaxin 1B from the plasma membrane and SNAP 23 from the acrosomal membrane. This *trans*-SNARE complex was not observed in control sperm.

**Conclusions/Significance:**

We propose that this capacitation driven membrane docking and stability thereof is a preparative step prior to the multipoint membrane fusions characteristic for the acrosome reaction induced by sperm-zona binding. Thus, sperm can be considered a valuable model for studying exocytosis.

## Introduction

Mammalian sperm cells are activated during migration in the female genital tract. This activation process (henceforth called capacitation) enables sperm cells to interact with the zona pellucida (ZP) of the oocyte which in turn triggers the acrosome reaction (AR) [1]. *In vitro*, this process can be mimicked by incubation of sperm cells in media containing bicarbonate/CO_2_ and albumin [2], [3]. Only capacitated sperm cells are able to undergo the zona-triggered AR and this process characteristically involves multipoint fusions of the sperm head plasma membrane (PM) with the outer acrosome membrane (OAM) [2].

Proteins from the Soluble NSF (N-ethylmaleimide-sensitive factor) Attachment protein Receptor (SNARE) family are widely regarded as key players in Ca^2+^-mediated membrane fusion processes [4]–[6]. Fusion in different cell types requires specific sets of SNAREs. SNARE-mediated exocytosis involves interactions of complementary SNARE proteins (typically Q-SNAREs in the plasma membrane and R-SNAREs in the vesicle membrane) to form 7S or 20S *trans*-SNARE protein complexes which after a calcium-dependent conformational change are believed to execute the membrane fusion [7]–[10]. The assemblage of the SNARE complex will not lead to an instant fusion, instead, the preformed complexes are stalled awaiting the appropriate stimulus (i.e. extracellular calcium influx) and additional factors (e.g. Rab 3A, NSF and complexin) which are involved in either the stabilization of the SNARE complex or the initiation of the actual fusion of two membranes [11]–[13]. It is clear that membrane docking (the first stage of membrane interactions in which complementary sets of SNARE proteins are brought and mixed together to form the SNARE complex) [4], [14], priming (the stage after the docking step that the formed *trans*-SNARE complex pulls the interacting membranes into a close and tight apposition) [4], [6], and fusion are distinct processes which are mediated by specific sets of proteins [14]–[16]. Multiple regulatory pathways and up/down stream cascades for SNARE complex formation have also been described [6]. Likewise in sperm, SNARE proteins have been identified and structural information regarding the ternary SNARE complex formation has been revealed recently, albeit that these analyses were carried out under calcium ionophore condition and/or under streptolysin-O permeabilization conditions [17], [18]. These treatments do not represent physiological conditions and as such do not only cause the formation of a *trans*-SNARE complex but also induce the fusion of interacting membranes (resulting in the formation of a *cis*-complex). These treatments also induce the loss of cell integrity. Until now it is not clear where and how SNARE proteins are physiologically involved in the regulation of acrosome docking, priming and fusion in sperm cells. Therefore, we studied for the first time how *trans*-SNARE complexes are formed and how the docking and priming of the acrosome membrane with the plasma membrane are regulated in living sperm cells upon capacitation. We mimicked physiological sperm capacitation by activating sperm with *in vitro* fertilization media and we assessed whether this treatment affected the docking and priming of the acrosome by formation of *trans*-SNARE complexes.

## Results

### Capacitation induces close apposition between the apical sperm head surface and the outer acrosomal membrane

**Figure 1:**
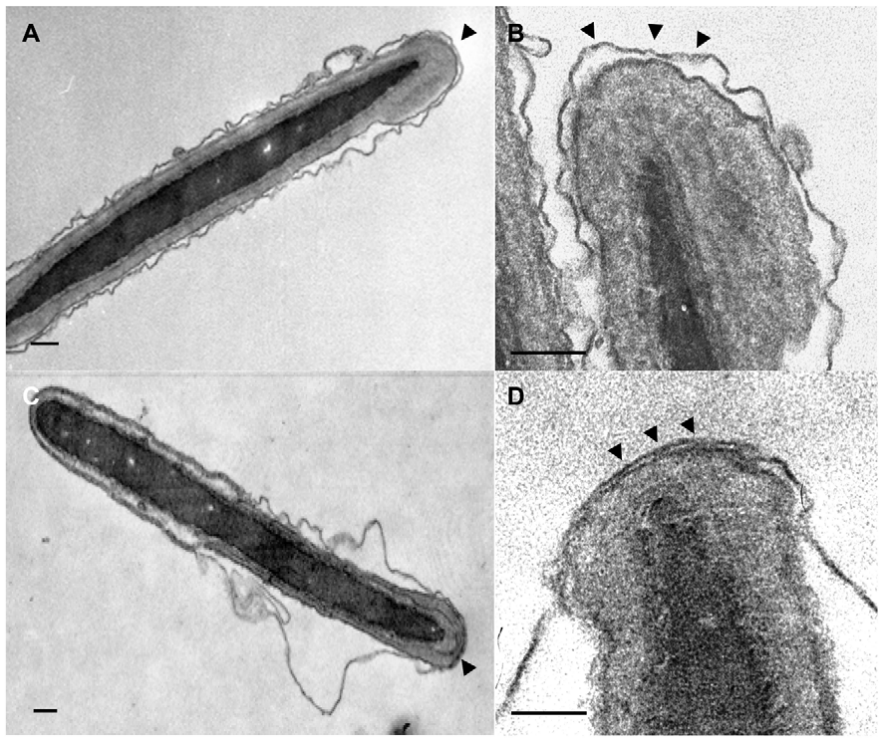
Capacitation alters the ultrastructure of the apical head and the acrosome of boar sperm. (**A**): Control non-capacitated boar sperm with a plasma membrane that is not tightly associated to the outer acrosomal membrane. The loose plasma membrane at the entire sperm head is due to processing of the specimen for TEM. (**B**): Higher magnification of the apical tip of the control non-capacitated sperm head. (**C**): The apical tip of a capacitated sperm head showing tight association of the plasma membrane with the outer acrosomal membrane whereas at a more distal area the two membranes do display a loose apposition (similar to control sperm). (**D**): Higher magnification of the apical tip of the capacitated sperm head. Arrow heads indicate the apical area of the sperm head. Bar represents 50 nm.

Ultrastructural examination revealed that uncavitated control sperm cells have a loose but continuous PM on the entire sperm head surface (Fig. 1A–B). This loose apposition is due to a lack of membrane interaction and the osmotic effects which occur during processing of the specimen for electron microscopy (EM) and indicates that the sperm acrosome is probably not docked with the PM in control sperm. Interestingly, sperm activation in an *in vitro* fertilization medium (capacitation) resulted in a close parallel arrangement of the apical PM with the underlying OAM in the uncavitated sperm cells (Fig. 1C–D). The PM in the non-apical area, on the other hand, showed the same loose arrangement (Fig. 1C–D) as was observed in control spermatozoa. This close apposition of the apical sperm head PM with its underlying acrosomal membrane (the two membranes are hardly distinguishable in that area) suggests that the two membranes are interacting with each other.

**Figure 2 pone-0011204-g002:**
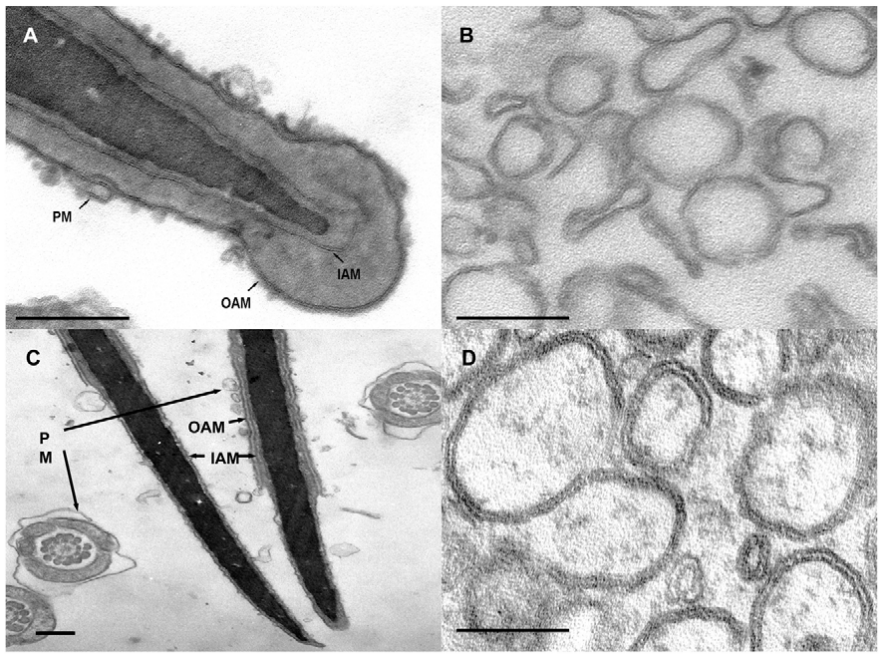
Capacitation promotes cavitation-induced release of membranes from the apical head and acrosome. (**A**): Cavitated control boar sperm heads show only the removal of apical plasma membrane after nitrogen cavitation. (**B**): Unilamellar membrane vesicles recovered from cavitated membrane fraction of control spermatozoa. (**C**): Capacitated boar spermatozoa show after cavitation that both plasma membrane and outer acrosomal membrane are removed at the apical area of sperm head while the plasma membrane overlying the area containing mitochondria remains intact and the outer acrosome membrane remains attached to the spermatozoa at the non-apical area of sperm head. (**D**): Bilamellar membranes structures emerge in cavitated membrane fractions from capacitated spermatozoa. PM  =  plasma membrane; OAM  =  outer acrosomal membrane; IAM  =  inner acrosomal membrane. Bar represents 200 nm.

**Figure 3 pone-0011204-g003:**
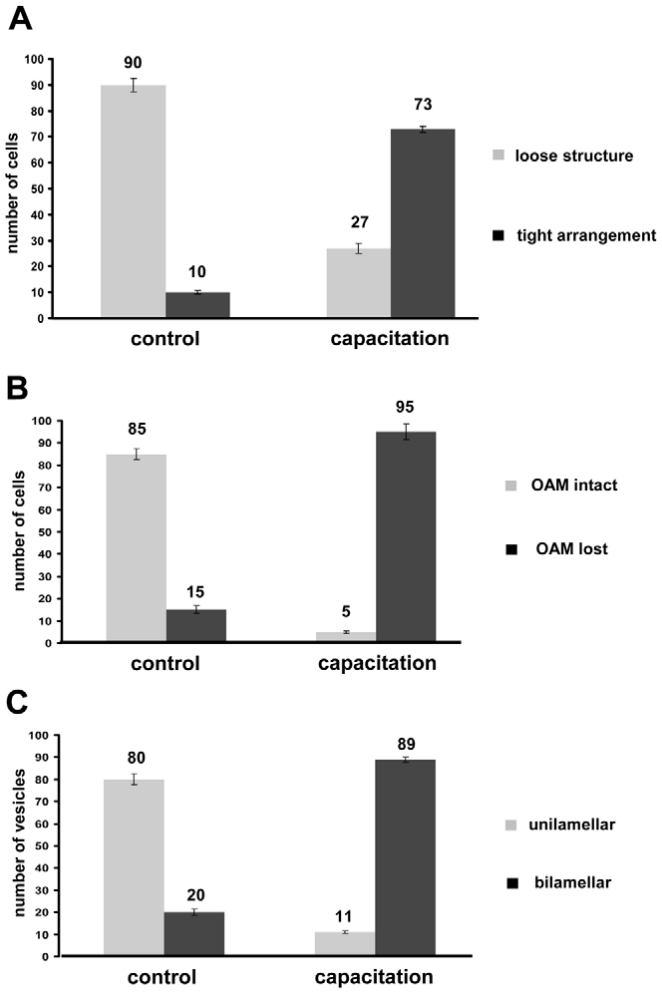
Quantification of PM and OAM arrangements of control and capacitated sperm and their corresponding cavitate fractions. (**A**) Quantification of the loose (gray) and tight (black) interacting PM and OAM (for morphologies see Fig. 1B and D respectively). (**B**) Quantification of intact OAM (gray) and removed OAM in the 1000 g cavitate (for morphologies see Figure 2A and C respectively). (**C**) Quantification of monolamellar (gray) and bilamellar (black) membrane structures in the 285000 g cavitate. For each experimental condition three independent sections were counted. Individual scores are provided (see Table S1 A–C) (n = 3 values are indicated ± SD for all the data presented the differences between capacitation and control conditions were significant; P<0.0001).

To test this possibility, we have subjected control and capacitated sperm to nitrogen cavitation which enables the separation of membranes at the apical area of the sperm head [19]. Control cavitated sperm showed that the 1000 g pellet consisted mainly of remaining sperm heads with intact acrosomes whereas the PM was released (Fig. 2A). The released apical PM (henceforth called the cavitated membrane fraction), resealed into unilamellar membrane vesicles that were recovered in purified form in the 285000 g pellet (Fig. 2B). In contrast, capacitated spermatozoa showed after cavitation and differential centrifugation, remaining sperm heads (in the 1000 g pellet fraction) with disrupted acrosomes at the apical sperm head area (i.e. where the two membranes were more closely attached in Fig. 1C–D). At that area, the cavitation procedure not only resulted in the release of the PM but also of the OAM (Fig. 2C). This may reflect a stronger interaction of the two membranes. This possibility is strengthened by the ultrastructural properties of the cavitated membrane fraction released from capacitated sperm. This membrane fraction characteristically showed a bilamellar morphology (i.e. with two interacting membrane bilayers; Fig. 2D) indicating to a stronger membrane interaction which remained intact after the cell disruption procedure and subsequent differential centrifugation steps during their isolation. In line with this, the more distal sperm head area, where the membrane interaction was not observed (Fig. 1C), did not show the stripping of the OAM (Fig. 2C). Furthermore, as observed in control sperm [19], the PM of the sperm tail and mid-piece remained attached to these structures after the cavitation treatment in capacitated sperm (Fig. 2C). Therefore, the isolated bilamellar membranes appeared to be specific for the sperm head area which is involved in the acrosome reaction and which was reported previously to be enriched in SNARE proteins after sperm capacitation [20]. The amount of sperm heads with (a) bilamellar versus no apposition of the apical PM with the OAM and sperm heads (Fig. 3A) (b) the amount of cavitated sperm heads with and without the apical OAM (1000 G, Fig. 3B) and (c) the amount of isolated unilamellar versus bilamellar membranes (285000 G, Fig. 3C) were quantified. Under control conditions <20% of sperm showed the close proximity of the PM and OAM whereas >73% (n = 3, P<0.0001) showed this after a 2 hours *in vitro* capacitation treatment (see also Table S1). We should stress here that the degree of spontaneous acrosome reaction during the incubation period for control and capacitated sperm was less then 3%, as has been reported previously (for review see [21] and see Fig. S1).

### Bilamellar membranes contain acrosomal components

In order to quantify the interactions between the OAM and the PM, we assessed the level of purity of the cavitate membrane fractions by measuring alkaline phosphatase activity (a marker enzyme for plasma membrane for boar spermatozoa) and of acrosin (a marker enzyme for acrosomal content for boar spermatozoa) [19].

Alkaline phosphatase activity in cavitate membrane fraction was respectively 10.6 and 11.7 times higher than in whole cell lysates for the capacitation or the control group which indicates that the purification of PM material was similar for both sperm treatment groups (Table 1). Under the control condition, only trace amounts of acrosin were detectable in cavitated membrane fractions (4% compared with whole cell lysate, Table 1), whereas significant increased acrosin activities were measured in the bilamellar membrane fractions from capacitated cells (Table 1). These results indicated that acrosomal material was co-purified due to disruption of the acrosome. Similarly, the OAM-specific lectin PNA (peanut agglutinin) binding site [19], [22] was enriched in the cavitated membrane fractions from capacitated sperm when compared to control sperm. The relative PNA-binding was significantly increased (approximately 74%) after capacitation treatment, indicating increased amounts of OAM in cavitated bilamellar membrane fractions (Table 1). The cavitated fractions were not contaminated with inner acrosomal membrane (IAM) since this membrane remained attached to the remaining sperm head (Fig. 2C) after cavitation and was completely separated from the vesicles during the differential centrifugation steps. Moreover, the cavitated membrane fractions were almost devoid of acrosin (an indicator of the presence of inner acrosome membrane [19], Table 1).

**Table 1:** Enrichment of acrosomal membranes in cavitation-released fractions from capacitated sperm.

Enzyme		Specific activity			Purification^a^
		whole sperm lysate	1000 g fraction	UCF (285000 g fraction)	
*Plasma membrane marker* b					
alkaline phosphatase	capacitated	7.5±0.6	8.8±0.7	79.2±7.3	10.6
	control	6.7±0.5	9.0±3.1	78.3±10.1	11.7
*Acrosomal marker*					
Acrosin^c^	capacitated	337.8±66.6	251.9±11.8	45.5±2.9^e^	0.1
	control	323.7±38.6	289.9±34.1	14.1±1.8^f^	0.04
*Outer acrosomal membrane marker*					
PNA-binding^d^	capacitated	509.2±64.9	279.0±13.5	126.6±14.1^g^	0.3
	control	504.4±58.3	228.4±30.1	72.6±16.9^h^	0.1

Alkaline phosphatase as a specific plasma membrane indicator for the purity of membrane isolates and acrosin as a marker for the level of acrosome contamination. Peanut Agglutinin (PNA) was used as marker lectin for the outer acrosome membrane material in the 285000 g fraction of the membrane isolates.

a ratio of the specific marker activities between UCF and whole sperm lysate.

b nmol • mg protein^−1^ • min^−1^.

c µIU: quantity of acrosin • µg protein^−1^ hydrolyzes 1 µmol BAPNA• min^−1^ at 22°C.

d Normalized OD_450_ • µg protein^−1^ • 10^9^ cell^-1^.

• 1000 g fraction contains sperm head

• UCF: Ultra-centrifuge fraction recovered from 285000 g pellet contain membrane vesicles.

• e-f, g-h: significant different for 2-tailed tests between IVF incubations (actual p values for tests between e-f and g-h are both <0.01).

• n≥5, ±s.d.

### Capacitation induces the formation of a ternary trans-SNARE protein complex

**Figure 4 pone-0011204-g004:**
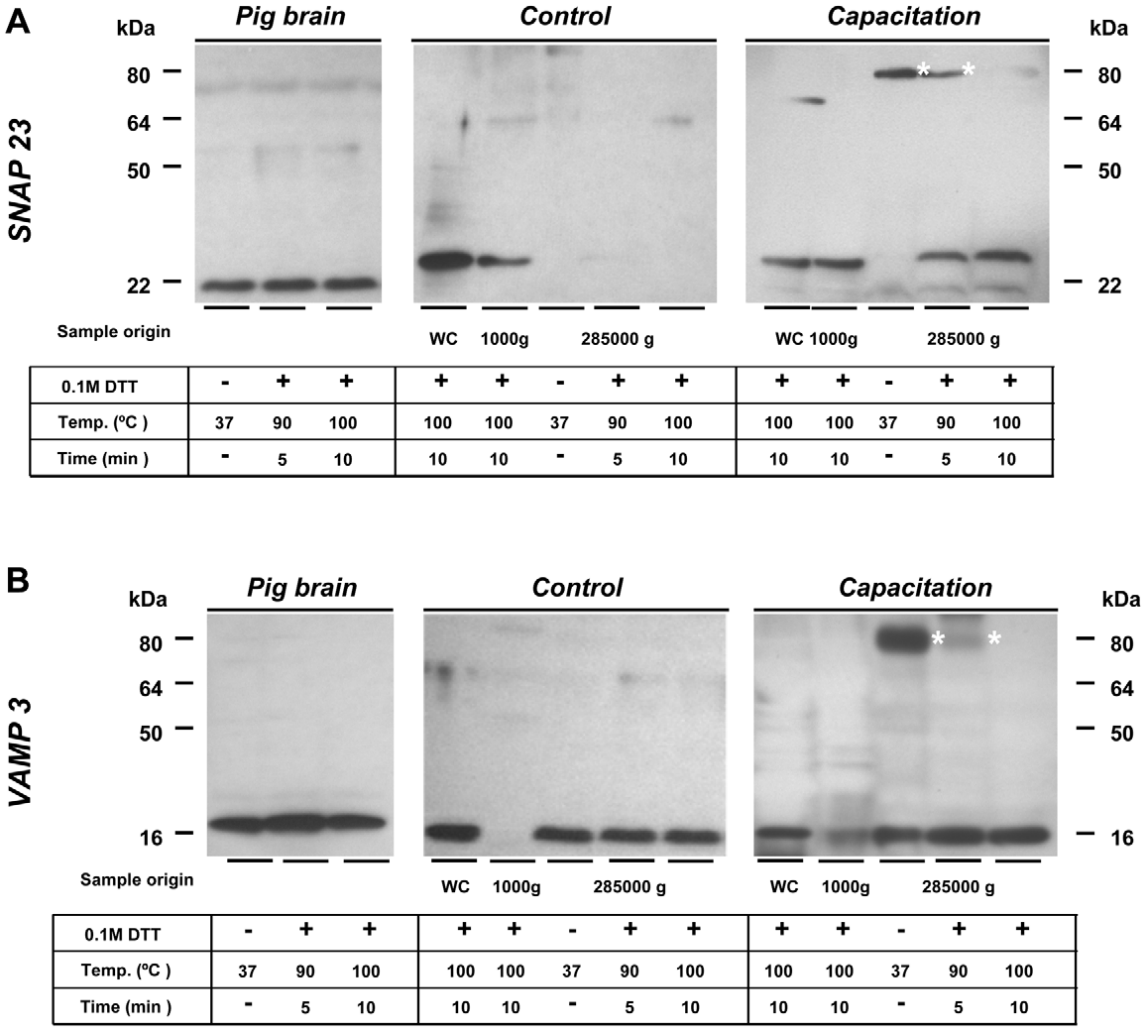
Capacitation-dependent formation of a ternary *trans*- SNARE protein complex. Cavitated membrane fractions (recovered from the 285000 g pellet) of control and *in vitro* capacitated sperms were isolated via nitrogen cavitation in combination with differential ultracentrifugation. The whole sperm lysate (WC) and the remaining sperm head (recovered from 1000 g pellet, without the apical membrane) under the same treatment were used for the comparisons for the presence of SNARE proteins/protein complexes. Samples including positive controls from pig brain were loaded under either non-reducing condition or treated with reducing agent (0.1 M DTT). The DTT samples were further heated at 90°C for 5 minutes or at 100°C for 10 minutes. (**A**): SNAP 23 appears in the free 23 kDa form in control sperm and in the capacitated whole sperm lysate and remaining head fraction (cavitate pellet at 1000 g). Interestingly, after capacitation the cavitated membrane fraction (pellet at 285000 g), contained a 80 kDa SDS-resistant SNAP 23-containing protein complex (marked with asterisks). This 80 kDa protein complex was resistant to the 0.1 M DTT and heat treatment up to 90°C but fully dissociated into the monomer SNAP 23 after 100°C heat treatment in presence of 0.1 M DTT. (**B**): VAMP 3 showed a plasma membrane-specific localization in the free 16 kDa form in control sperm. SDS and partially thermal and 0.1 M DTT resistant VAMP 3-containing SNARE complexes can be detected at 75–80 kDa after sperm capacitation (marked with asterisks). Also this protein complex fully dissociated into monomeric VAMP 3 at 16 kDa under reducing conditions with 0.1 M DTT for 10 minutes at 100°C. 10 µg of total protein extract was used for all samples. Pig brain homogenate was used as positive control with comparable experimental setups. WC =  whole cell lysate; 1000 g =  remaining cavitated head fraction; 285000 g  =  cavitated membrane fraction.

Western-blotting showed that the Q-SNARE protein SNAP 23 was present at a molecular weight (MW) position of 23–25 kDa in remaining sperm heads in both capacitated and control sperm (Fig. 4A). SNAP 23 was not present in the cavitated membrane fraction of control sperm (Fig. 4A); however, a substantial signal for SNAP 23 emerged in the cavitated membrane fraction of capacitated sperm (Fig. 4A) at a 80 kDa band under the non-reducing condition. These SNAP 23-containing protein complexes partially dissociated after a heat treatment at 90°C and fully dissociated into monomers after heat treatment at 100°C. The abundance in the higher MW SNAP 23 found in the boar sperm (when compared with boar brain) might be attributed to tissue specific differences. The presence of SNAP 23 in the cavitated membrane fraction of capacitated sperm as well as its emergence in a high MW band was unexpected. Therefore, we performed a series of experiments to determine which SNARE proteins (e.g. syntaxin and VAMP) interacted with SNAP 23 upon capacitation. Among the SNARE proteins tested, the R-SNARE protein VAMP 3 appeared as a 16 kDa band in the control cavitated membrane fraction (Fig. 4B). Note that there is a low (16 kDa) molecular weight form predominant in sperm and a high (18 kDa) molecular weight form predominant in brain (Fig. 4B). The differences in low and high molecular weight forms are probably cell specific expression variability [23]. After capacitation, VAMP 3 appeared in a similar capacitation-dependent 80 kDa band as was seen for SNAP 23 (Fig. 4B). Heating these samples at 100°C with 0.1 M DTT caused the dissociation of the 80 kDa protein complex and the appearance of VAMP3 as a 16 kDa monomer band (Fig. 4B).

**Figure 5 pone-0011204-g005:**
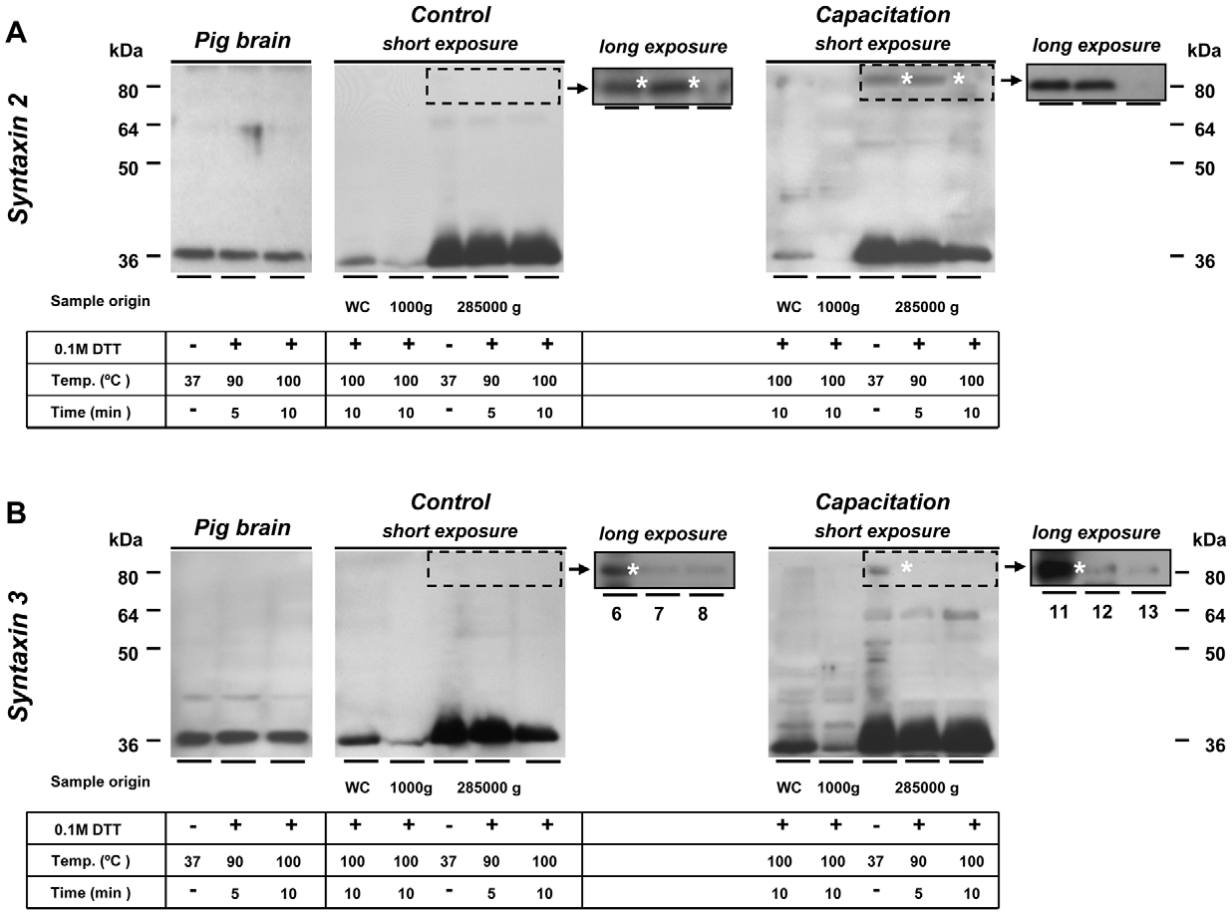
Syntaxin 2 and 3 interact with additional proteins and do so partly independent from capacitation. (**A**): Syntaxin 2 was enriched in the cavitated membrane fraction (285000 g pellet) in the free 36 kDa form in both control and capacitated sperm cells. A weak signal at 80 kDa was observed in control cavitated membrane fractions (long exposure blot, marked with asterisks); this 80 kDa protein band was enhanced upon capacitation indicating a capacitation-enhanced but not capacitation-dependent interaction of syntaxin 2 with other proteins. (**B**): Syntaxin 3 also showed capacitation-independent formation of a 80 kDa SNARE-containing complex and this interaction (formation) is promoted by capacitation. However, due to the enrichment of both syntaxins in the cavitated membrane fractions and the relative small amount of complexed syntaxins (∼15%, see [20]) compared to the uncomplexed monomeric syntaxin, the dissociation of the syntaxins can not be observed clearly in these samples. 10 µg of total protein extract was used for all samples. WC =  whole cell lysate; 1000 g =  the remaining cavitated head fraction; 285000 g =  cavitated membrane fraction.

Whole sperm lysates and cavitate membrane fractions contained syntaxin 2 and 3, which were detected as a monomeric 36 kDa band in both capacitated and control sperm. Both syntaxins were enriched in the PM when compared to the acrosomal membrane (the remaining head fraction Fig. 5A–B). Similar to SNAP 23 and VAMP 3, both syntaxins also appeared in a heteromeric 80 kDa protein band after capacitation. The capacitation treatment resulted in an increase of syntaxin2/3-containing 80 kDa heteromeric complex (Fig. 5A–B, long exposure). However this occurred only to a marginal fraction of syntaxin 2 and 3 while the predominant fraction remained in its monomeric 36 kDa form. Heat treatment of the cavitated membrane fractions caused a disruption of the 80 kDa syntaxin 2/3-containing complex into monomers. We should note that 90°C heat treatments also resulted in incomplete dissociation of the 80 kDa band into a 65 kDa band that was positive for syntaxin 2/3 (Fig. 5B).

### Syntaxin 1B is the cognate Q-SNARE with SNAP 23 and VAMP 3

**Figure 6 pone-0011204-g006:**
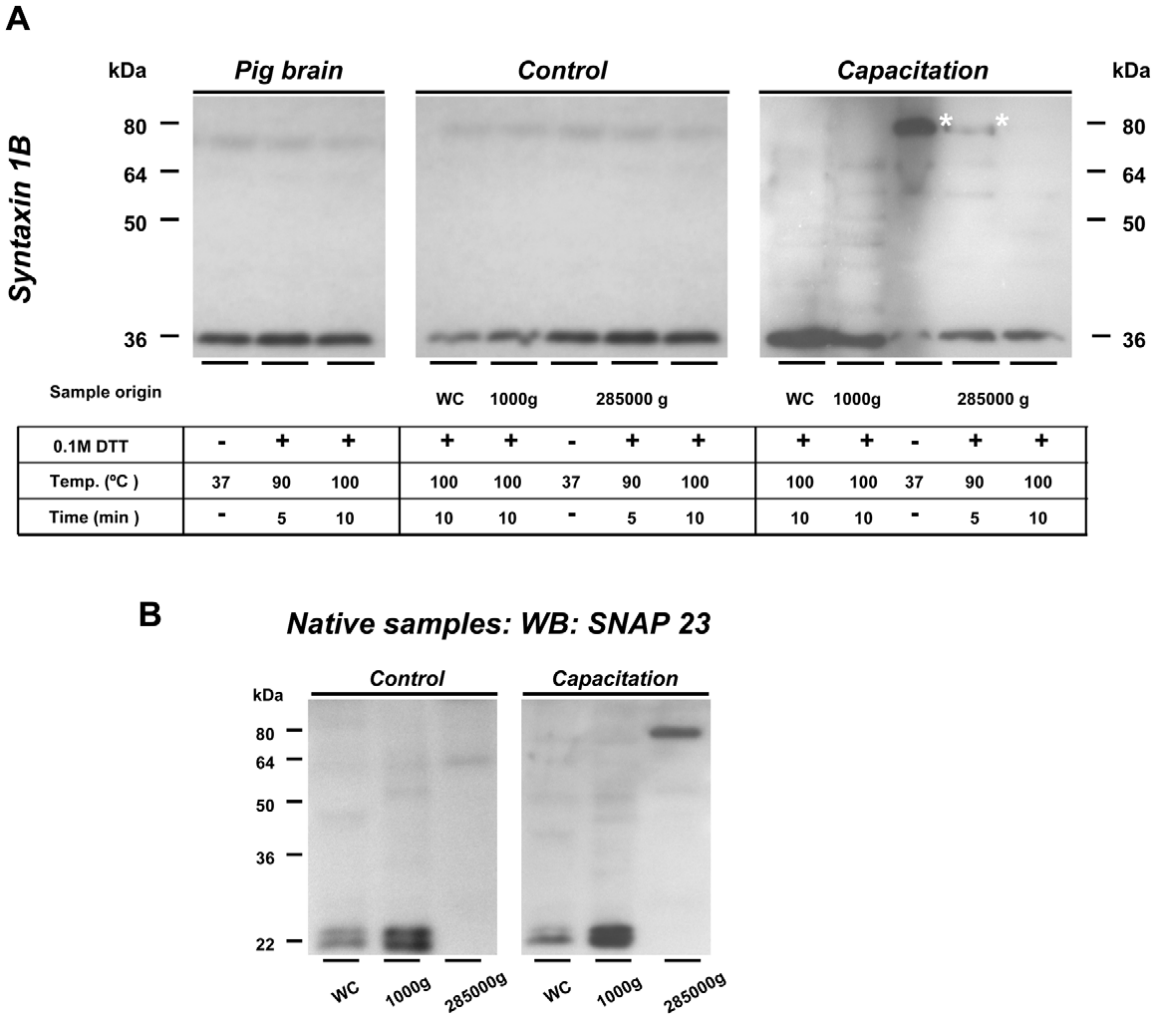
Syntaxin 1B is the interacting Q-SNARE partner of SNAP 23 and VAMP 3 upon capacitation. (**A**): In control sperm syntaxin 1B appeared predominantly in the cavitated membrane fraction (285000 g) but also in the remaining cavitated sperm head fraction (1000 g) in the free 36 kDa form. However, a 80 kDa SDS-resistant syntaxin 1B-containing protein complex emerged in the non-reducing cavitated membrane fraction of capacitated sperm cells indicating its capacitation-dependent interaction with other proteins. This 80 kDa protein complex shows the same 0.1 M DTT and thermal stability (up to 90°C) as the SNAP 23 and VAMP 3-containing protein complexes (Figs. 4). Experimental set ups were identical as in Figs. 4 and 5. (**B**): Western blot data of non-reducing samples revealed that the presence of a 80 kDa protein complex is exclusive for the cavitated membrane fraction from capacitated sperm (285000 g pellet). WC =  whole cell lysate; 1000 g =  remaining cavitated head fraction; 285000 g =  cavitated membrane fraction. 10 µg of total protein extract was used for all samples. Pig brain homogenate was used as positive control.

Participation of syntaxin 2 and 3 in a 80 kDa SNARE complex in control sperm was in contrast to that of reported for VAMP 3 and SNAP 23 (see above section). Moreover, the syntaxin 2 and 3-containing 80 kDa complex showed different thermal stability when compared to VAMP 3 and SNAP 23-containing 80 kDa complex. We therefore investigated the presence of another syntaxin isoform (syntaxin 1B) in sperm, and whether this syntaxin was responsible for the capacitation-specific formation of the VAMP 3/SNAP 23-containing 80 kDa SNARE complex. The rationale for this is that we previously showed that syntaxins 1–3 display a similar redistribution behavior to the apical ridge of the sperm head upon capacitation [20], and that syntaxin 1 has been reported to interact with SNAP 23 [24] and VAMP 3 [25], [26]. Our data showed that syntaxin 1B was present in sperm and it only appeared in the monomeric form (36 kDa, Fig. 6A) in control sperm. Almost all syntaxin 1B participated in the capacitation-dependent formation of the 80 kDa heteromeric SNARE complex. This complex was only recovered in the cavitated membrane fraction (Fig. 6A). The complex was stable under non-reducing condition, but completely dissociated into monomeric syntaxin 1B (36 kDa) after 100°C heat treatment (Fig. 6A). Remarkably virtually no SNARE complex dissociation was seen when the samples were treated with either 0.1 M DTT at 37°C or after a 10 minute at 100°C in absence of DTT (Fig. S2) while SNARE complexes were not detected in pig brain homogenates. The OAM-specific SNAP 23 only participated in the 80 kDa SNARE complex recovered from cavitated bilamellar membrane fraction of capacitated sperm. The 80 kDa SNAP 23 containing complex was not present in other sperm sub-fractions (Fig. 6B). This was indicative for the capacitation-dependent formation of a *trans*-SNARE complex between OAM and PM: SNAP23 was only present in the OAM (Fig. 6B, lanes for control sperm) while VAMP 3 was exclusively localized in the apical PM (Fig. 4B lanes for control sperm). Thus despite the fact that syntaxin 1B is not exclusively localized in the PM but also resides in the cavitated 1000 G fraction of control sperm (Fig. 6A lanes for control sperm) we have no other explanation for the formation of the SNARE complex than the actual docking of the PM with the OAM by *trans* SNARE complex formations. The formation of *trans*-SNARE complexes also explain the capacitation dependent close bilamellar arrangement of PM and OAM (Figs. 1–3).

### Co-immunoprecipitation reveals SNARE protein interactions upon capacitation

**Figure 7:**
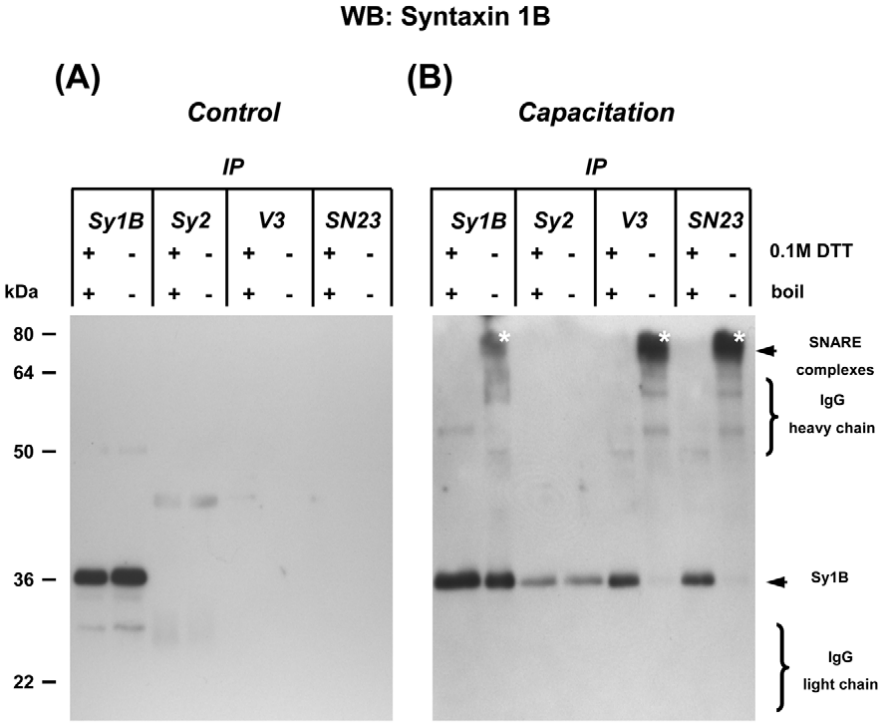
Detection of capacitation-dependent SNARE interactions by co-immunoprecipitation. Cavitated membrane fractions (285000 g) from boar sperm were used. Samples were treated in non-reduced condition or in full-reduced condition that were heated at 100°C for 10 minutes. (**A**) No SNARE interactions can be observed in the control cavitated membrane fractions. (**B**): SDS-resistant SNARE complexes emerged after capacitation when IP was performed with either anti-VAMP 3 or anti-SNAP 23 antibodies indicating the trimeric interactions between syntaxin 1B/SNAP 23/VAMP 3. The noted 80 kDa protein complex further substantiates the observed capacitation-dependent formation of ternary *trans*-SNARE complex in Western-blot experiments. This 80 kDa protein complex fully dissociated into the constituted SNARE monomers under 100°C heat treatment in the presence of 0.1 M DTT and can be detected at 36 kDa when WB was performed subsequently with anti-syntaxin 1B antibody. Syntaxin 2 is not expected to have specific interaction with syntaxin 1B and is therefore used as additional negative control to demonstrate the specificity of the detected signals.

Co-immunoprecipitation (IP) experiments were performed to reveal the possible capacitation-dependent interactions of SNARE complex partner proteins (i.e. syntaxin 1B, SNAP 23, VAMP 3) during acrosome docking and priming. When control cavitated membrane fractions were used, only a 36 kDa signal was detected after IP with syntaxin 1B (Fig. 7A). This is in line with Fig. 6A where Western-blot experiments using anti-syntaxin 1B antibodies indicated that both SNAP 23 and VAMP 3 did not interact with syntaxin 1B in control sperm. Two controls were performed to show the specificity of the IP results: (1) Bead labeling was performed with IP antibody together with a two fold amount of control rabbit IgG (this gave similar results as depicted for Fig. 7A, data not shown), (2) Bead labeling with only control rabbit IgG which did not result in any positive bands (data not shown). IP with anti-SNAP 23 and anti-VAMP 3 antibodies with the cavitated membrane fraction from capacitated sperm resulted in a SDS-resistant protein complex which contained SNAP 23/VAMP 3/syntaxin 1B under non-reducing condition at 80 kDa (Fig. 7B). This protein complex dissociated into the monomeric SNARE proteins when the samples were treated with 0.1 M DTT during 10 minutes 100°C for example the 36 kDa band of syntaxin 1B as detected by Western blot (Fig. 7B). The presence of the 80 kDa SNARE-containing protein complex demonstrates the trimeric interactions between syntaxin 1B/SNAP 23/VAMP 3 upon capacitation. The detected 80 kDa SNARE protein complex further confirmed the formation of the *trans*-SNARE complexes upon capacitation required for stable acrosome docking (Fig. 1–6).

## Discussion

Mammalian sperm must acquire the ability to fertilize the oocyte after ejaculation and this process is called ‘capacitation’ [1]. Capacitation of sperm cells involves the activation of several signalling pathways such as sperm specific adenylyl cyclase, cAMP-dependent protein kinase A (PKA) and protein tyrosine phosphorylation [2] as well as reorganization of proteins and lipids on the plasma membrane [21]. Proteins belonging to the SNARE family have been shown to regulate sperm exocytosis and functional studies have provided evidence for a possible role in fertilization [17], [18], [27]. Although several studies have showed the importance of SNARE interactions in the modulation of sperm acrosome exocytosis (also known as the acrosome reaction), no information is available on the spatial-temporal rearrangements of these protein interactions during capacitation. With this respect, it is of interest to mention that the classical topologies of SNARE proteins in somatic cells on target membrane and vesicle membrane is probably more complex in sperm. This may have to do with the fact that the Golgi apparatus rolls over the growing acrosomal granule during acrosome formation in developing spermatids and exposes its *trans* Golgi side towards the acrosome granule and its *cis* side towards the plasma membrane [28]. This largely neglected Golgi reorganization phenomenon in spermatids may cause the noted aberrant topology of SNAP 23 (at the outer acrosomal membrane) and VAMP 3 (at the plasma membrane) when compared to somatic cell types.

Previously, we have demonstrated that both Q- and R-SNAREs (also known as t- and v-SNAREs) show a bicarbonate- and albumin-dependent redistribution to the apical area of the sperm head upon capacitation [20]. In the present work, we have expanded our studies to establish whether this bicarbonate- and albumin-dependent redistribution of SNARE proteins underlies the formation of *trans*-SNARE complexes resulting in stable docking and priming of the plasma membrane, and the acrosomal membrane, without the emergence of the spontaneous acrosome reaction. The stabilization of this 80 kD SNARE complex probably serves to prevent AR. Indeed, only minimal amount of sperm showed spontaneous AR under control and capacitation conditions (Fig. S1).

Our ultrastructural data showed that capacitation induces a close apposition of the apical PM with the OAM. The two membranes could be isolated from sperm by means of nitrogen cavitation and differential centrifugation steps. These cavitated membrane fractions showed a stable bilamellar membrane structure. In control sperm, the same cell disruption technique resulted in unilamellar vesicles representing the apical plasma membrane only. These results suggest that sperm capacitation induces the interactions of the acrosome membrane with the apical sperm head PM.

Analysis of membrane specific enzyme activities in the cavitated membrane fraction confirmed this speculation as cavitated membranes from capacitated sperm contained 74% more PNA-binding sites (indicative for the OAM [19], [22]) when compared to control sperm cavitated membrane fractions. The efficiency of apical PM isolation from control and capacitated sperm were quantitatively and qualitatively similar and were only minimally contaminated with other sperm components. Western-blot studies showed that the acrosomal SNAP 23 was only present in the cavitated membrane fraction after capacitation and appeared in a partially heat-resistant 80 kDa form and is specific for the OAM. VAMP 3, in contrast to other isoforms of VAMP (namely VAMP1 and VAMP2; [20]), is specific for the PM and only present in monomeric form in control sperm emerged predominantly in 80 kDa band in capacitated sperm. Three syntaxins (1B, 2 and 3) are predominantly present in the sperm PM (the cavitated control sperm membrane fraction) but were also present in the remaining control cavitated sperm head fraction. Syntaxin 2 and 3 (unlike SNAP 23 and VAMP 3) were predominantly present in a monomeric 36 kDa form. Only a small fraction of syntaxin 2/3 was already present in the 80 kDa protein complex and only a marginal increase of these complexes were found after capacitation while still most syntaxin 2 and 3 remained detectable in their monomeric form. Therefore, these two syntaxin isoforms are probably are not the primary cognate Q-SNARE with SNAP 23 and VAMP 3. In stead, the presence of syntaxin 1B in the 80 kDa SNARE complex formed under capacitation as well as its SDS- and thermal-stability characteristics suggest that syntaxin 1B/SNAP 23/VAMP 3 are the interacting SNARE proteins in the 80 kDa *trans*-SNARE complex. The involvement of the OAM-specific SNARE protein SNAP 23 and the PM specific VAMP 3 in the 80 kDa protein complex of cavitated membrane fractions provided evidence that capacitation induces the docking of the acrosome membrane to the PM. The exclusive presence of 80 kDa SNARE complex in the capacitated cavitated membrane fraction supports the possibility of SNARE-mediated docking and priming of PM and OAM. A role of the syntaxin 1B/SNAP 23/VAMP 3 complex in the regulation of exocytosis has been reported for other cell types [25]. Moreover, the conformational switch of a specific syntaxin isoform (i.e. syntaxin 1B) is important to control the synaptic vesicle fusion [8].

SNARE interactions appear to be non-selective [29] and thus could hypothetically lead to various SNARE protein combinations. Hence, we expect more than one type of SNARE complexes in sperm cells which may explain the additional detection of syntaxin 2/3-containing SNARE protein complexes. Furthermore, different SNARE complexes in cells could play a role on the modulation of exocytosis as described in human neutrophils [30]. The various combinations of SNARE proteins may to some extent, explain the unexpected syntaxin 2 and 3-containing SNARE complexes found in both control and capacitated sperm cells. We observed that both VAMP 1 and VAMP 2 participated in protein complex formation independently from capacitation (Fig. S3), which suggests that syntaxin 2/3 and VAMP 1/2 could be the cognate interacting SNARE proteins and form an additional pool of SNARE complexes. The preformed SNARE complexes in control sperm likely do not participate in PM docking to the OAM as these two membranes were easily separated by cavitation. The exact role of existing and additional formed syntaxin 2/3 SNARE complexes and their role as well as the role of VAMP 1/2 containing SNARE complexes in the regulation of the acrosome reaction remains to be elucidated.

SNAP 25 was not found in any of our sperm preparations (data not shown), which is in line with the observation that SNAP 23, an ubiquitously expressed homolog of SNAP 25, is present in non-neural tissues and is able to bind different syntaxins and VAMP isoforms [24]. In contrast to Yang et al. who showed that SNAP 23-containing SNARE complexes have an on average lower temperature resistance (71–83°C) [29], we found in this study stable SNAP 23-containing SNARE complexes with a higher thermal stability (up to 90°C). Therefore sperm capacitation, together with the different combinations of SNARE proteins, can cause variability in the stability of SNARE complexes.

Interestingly, SNAP 23 shows a three times higher raft/detergent resistant membrane (DRM)-association affinity when compared with SNAP 25 [31]. We have shown in a previous study that SNARE proteins cluster into raft membrane areas upon capacitation [20] and it is therefore possible that they gain extra protection from these large functional DRMs to withstand the changes (i.e. temperature, pH etc.) in the surrounding environment in order to assure the formation of stable SNARE complexes that can lead to a successful AR upon calcium stimulation. The idea -supported by the group of Boekhoff - is that a lipid raft associated scaffold protein is controlling the acrosome reaction in mammalian spermatozoa [32], [33] and as such may serve to direct the acrosome and plasma membrane SNAREs into the right membrane topology for the formation of stable ternary complexes. In fact the same group recently reported that CaMKIIalpha interacts with multi-PDZ domain protein MUPP1 in spermatozoa to prevent spontaneous acrosomal exocytosis [34].

Future research should emphasize whether and how this interaction as well as our previously reported emergence of SNARE proteins into surface membrane rafts in capacitation sperm relate to the capacitation-dependent docking of the acrosome to the plasma membrane and that this docking does not resulting in the acrosome reaction. In this light it is of considerable interest to note that bilamellar membrane structures that are formed after sperm capacitation as well as the formed *trans-*SNARE complexes –specific for these bilamellar structures- remain stable after their post-cavitation isolation. In fact the SNARE complex is resistant to high temperature and to reducing conditions and only falls apart after into SNARE monomers after a combinational treatment of reducing agents and high temperature. Probably, this higher *trans* SNARE complex stability compared to somatic cell types [29] relates to our observations that the efficient acrosome docking did not result in acrosome fusions involved in the AR.

In other cell types, SNARE proteins have been suggested to assemble into SNARE complexes prior to actual fusion of membranes [15], [35]. The observed ternary *trans*-SNARE complex formation is in line with studies on streptolysin-O permeabilized studies on mouse sperm [33] and on see urchin sperm [36]. In both species, these SNARE interactions were functionally linked to the calcium-dependent acrosome reaction. In similarity to other cell types, other factors like Rab 3 and complexin are most likely involved in formation of the observed ternary *trans*-SNARE complexes [37]–[39]. The functional relevance of these SNARE interacting/associating proteins on the regulation of acrosome reaction is currently under investigation.

**Figure 8:**
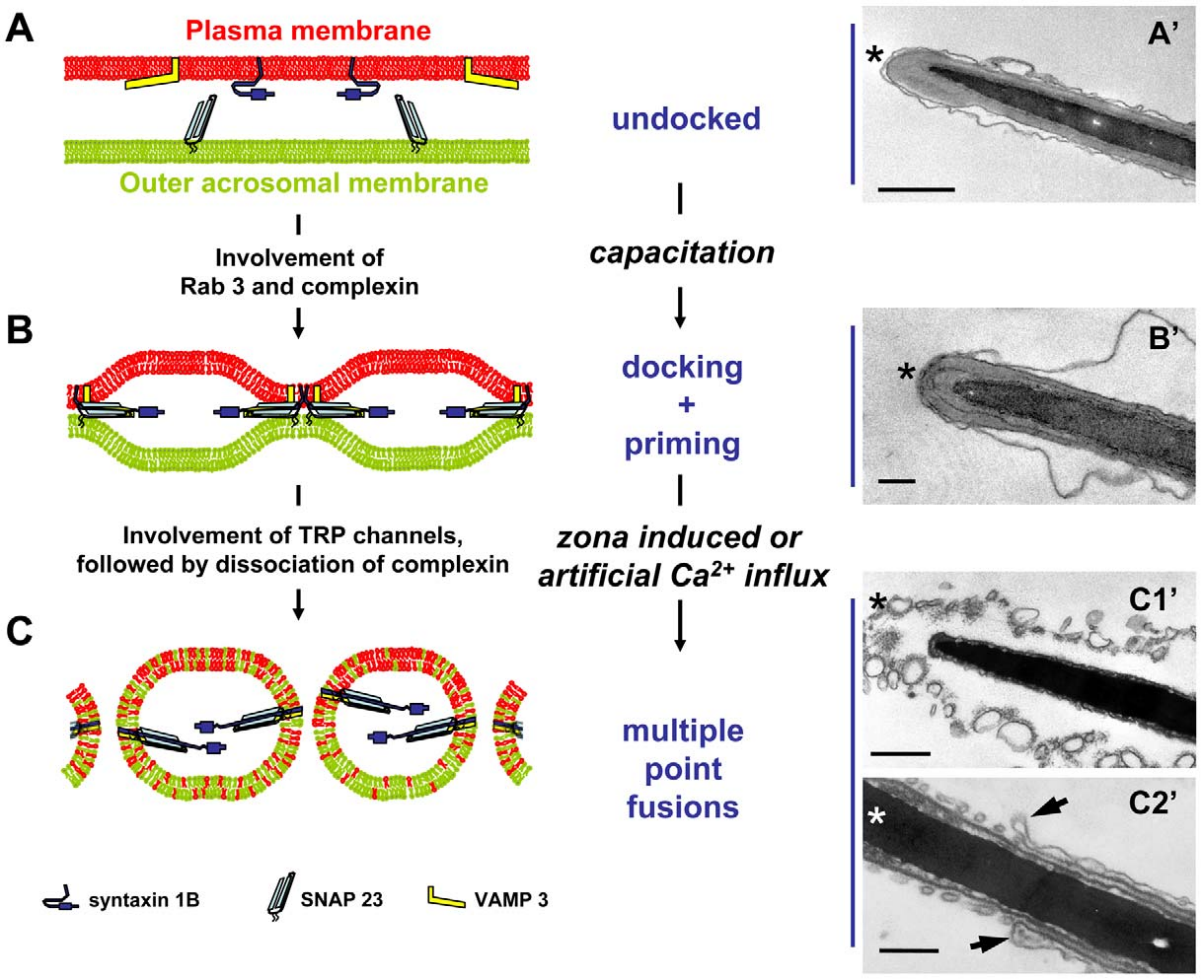
Hypothetical model for capacitation-induced stable docking of the acrosome to the sperm plasma membrane. (**A**): In control sperm the PM (in red) and OAM (in green) are not associated together. We found syntaxin 1B (purple blue) and VAMP 3 (yellow) at the PM and SNAP 23 (light blue) at the OAM. The undocked PM appears as loose arrangement at the entire head area due to the osmotic effect by EM processing (A′). (**B**): *In vitro* capacitation caused stable docking of the OAM to the apical PM. A stable ternary- SNARE complex is formed but this did not result in exocytotic membrane fusions (B′). (**C**): Exocytotic membrane fusions are executed after sperm binding to the zona pellucida or *in vitro* by calcium ionophore treatments. Mixed vesicles of the apical PM and the OAM are the result of the multipoint fusions characteristic for sperm acrosome exocytosis (C′1). However, at the equatorial sperm head area (distal from the arrows indicated in panel C′2) this fusion does not take place (C′2). This sperm surface area is specifically involved in the later occurring adhesion and fusion processes between the sperm cell and the oocyte leading to fertilization. The asterisks indicate the apical side of the sperm head. Bar represents 50 nm.

We here show that the stable capacitation-dependent docking of OAM to PM is not accompanied by membrane fusions. Instead, after docking and priming, the bilamellar structure remains intact and the two interacting membranes persist together as bilamellar membrane structure as discussed above. This implies that the formation of ternary *trans*-SNARE complexes is not sufficient to induce the AR but require additional stimuli or the removal of inhibitory components to allow the fusion reaction. Thus we may conclude that the physiological acrosome reactions are not identical to the calcium ionophore-induced acrosome reaction. Calcium ionophore treatments have recently been shown to induce multiple point fusions of the PM with the OAM [40]. The main difference between capacitated sperm and ionophore treated sperm is that acrosome fusions are not initiated by the capacitation treatment (this study) but do occur after ionophore treatment. Consequently, we propose that the working model for the acrosome reaction from Roggero et al. [38]: *(i)* the introduction of extracellular calcium between the acrosome and plasma membrane by ionophore treatment that caused formation of loose *trans*-SNARE complexes and *(ii)* an intra-acrosomal calcium efflux causing the execution of the SNARE-mediated acrosome exocytosis, should be refined as suggested in our model depicted in Fig. 8. We propose now that: (1) the acrosome is not associated with the plasma membrane in freshly ejaculated (control) sperm; (2) sperm capacitation leads to stable docking of the two membranes at the apical part of the sperm head but this occurs without the execution of acrosome fusions; (3) ZP binding allows Ca^2+^ entry into the sperm [41], [42] and the -as a consequence- elevated cytosolic calcium levels will evoke a calcium sensitive conformational change of the *trans-* to *cis-*SNARE complex. This is accompanied by the execution of the membrane fusions involved in the acrosome reaction. The involvement of SNARE proteins in these membrane fusions have been established by other groups [17], [27]. Novel is the capacitation-dependent transition from step A to step B (Fig. 8), which essentially summarizes the merits of the current study.

We propose that zona binding is required for a sufficient increase in free calcium to execute the fusions of the PM with the acrosome [41] which probably involves the opening a transient receptor potential (TRP) channel allowing transiently increased intracellular calcium levels [43]. In this light, it is interesting that the entire area where the docking and priming of the PM and the acrosome takes place is the same area where the sperm cell initially will bind to the zona pellucida [1], [2]. Moreover, this area coincides with the area where sperm membrane rafts have been shown to aggregate during capacitation treatments [20], [44], [45]. The stability and extended area of these docked and primed membranes may explain why after zona binding, the acrosome reaction is executed with its characteristic feature of multiple membrane fusions. These interactions between the two membranes involved in the acrosome reaction and the actual fusions of these membranes as result the generation of mixed vesicles (Fig. 8C1′–2′) are therefore, physiologically separated processes in mammalian fertilization.

In conclusion, we have shown that SNARE proteins form stable ternary *trans*-SNARE complexes during sperm capacitation. As a result of this interaction, the acrosome becomes stable docked to the apical sperm head plasma membrane without the occurrence of fusions between the two interacting membranes. The remarkable segregation of membrane docking and fusion events as well as the extended surface area where both processes take place around the acrosome reaction makes sperm suitable as model for studying ultrastructural and biochemical aspects of exocytosis.

## Materials and Methods

### Reagents and antibodies

Chemicals were obtained from Merck (Darmstadt, Germany), unless otherwise stated. Rabbit polyclonal antibodies against syntaxin 1B, 2, 3, SNAP 23, VAMP 3 (specific for this isoform) were obtained from Synaptic Systems (Göttingen, Germany). Immunoprecipitation reagents ReliaBLOT™ and Exacta Cruz™ F were purchased from Bethyl Laboratories, Inc. (Montgomery, TX, USA) and Santa Cruz Biotechnology (Santa Cruz, CA, USA), respectively. Biotin conjugated peanut agglutinin (Biotin-PNA) was from EY laboratory (San Mateo, CA, USA). Horseradish peroxidase (HRP) conjugated to streptavidin and 3,3′,5,5′-tetramethylbenzidine (TMB) were obtained from Sigma (St Louis, MO, USA). EDTA free protease inhibitor was obtained from Roche (Mannheim, Germany). Boar brain tissue homogenate was prepared as previously described [20] and used as positive control.

### Semen preparation and isolation of membrane vesicles

Freshly ejaculated sperms from highly fertile *Sus scrofa domestica* (pig) were obtained from commercial breeder (Cooperative Center for Artificial Insemination in pigs, ‘Utrecht en den Hollanden’, Bunnik, the Netherlands). All solutions were iso-osmotic (300±5 mOsm/kg) at room temperature (RT) and with protease inhibitor. Sperm was subjected to two *in vitro* fertilization (IVF) conditions; *capacitation* and *control* condition as previously described [20]. In short, sperm cells were incubated in HBT (HEPES buffered Tyrode's medium: 90 mM NaCl, 21.7 mM lactate, 20 mM HEPES, 5 mM glucose, 3.1 mM KCl, 1.0 mM pyruvate, 0.4 mM MgSO_4_, 0.3 mM NaH_2_PO_4_, 2 mM CaCl_2_, 100 µg/ml kanamycine; 300 mOsm/kg, pH 7) either supplemented with 15 mM NaHCO_3_ in open vials for 2 hours at 38.5°C in humidified atmosphere with 5% CO_2_ (capacitation) or incubated in HBT medium with the omission of bicarbonate in air-tight vials for 2 hours at 38.5°C in a tube rack placed in a pre-warmed water bath (control). Both conditions were prepared in the presence of the cholesterol acceptor bovine serum albumin (0.3% w/v BSA: essential fatty acid free, delipidated fraction V; Boehringer, Mannheim, Germany). Routinely, sperm were screened for cell integrity and spontaneous acrosome reactions using a flow cytometric protocol described previously [44].

For membrane vesicles isolation, a standard nitrogen cavitation protocol was followed [19]. One billion Percoll-washed sperm cells in TBSS buffer (Tris buffered sucrose solution; 5 mM Tris, 250 mM sucrose, pH 7.4) were subjected to nitrogen cavitation in a cell disruption device (Parr Instruments, Moline, IL, USA). The cavitate was slowly extruded and centrifuged at 1000 g for 2 times 10 min at 4°C. Supernatants were combined and centrifuged at 4°C for 10 min at 6000 g. Supernatant from 6000 g centrifugation was carefully loaded on top of a 50 µl, 80% (w/v) sucrose layer to prevent the stickiness of membrane materials at the bottom of the tube. Membrane materials were pelleted by ultracentrifugation at 4°C (2 times 70 min at 285000 g). Membrane pellets were re-suspended in HBS (HEPES buffered saline; 5 mM HEPES, 2.7 mM KCl, 146 mM NaCl, pH 7.4). Purity of the membrane isolates were assessed by measuring the alkaline phosphatase and acrosin activities [19], [46]. The remaining sperm heads (pellets that resulted from the 1000 g centrifugation described above), sperm tails (pellets that resulted from the 6000 g centrifugation described above) and the cavitated membrane fraction were flash-frozen in liquid nitrogen and stored at −20°C for later use.

### Transmission electron-microscopy (TEM)

Samples used for TEM purpose were recovered from Percoll-washed, cavitated or uncavitated spermatozoa. Cavitated membrane fractions (recovered from 285000 g ultra-centrifugation) under two IVF treatments were pelleted. Both samples were fixed overnight at 4°C in Karnovsky (contains 2% (v/v) paraformaldehyde and 2.5% (v/v) glutaradehyde diluted in cacodylate buffer) fixative. Pellets were washed with 0.1 M Na-cacodylate (pH 7.4) and post-fixed with 1% osmium tetraoxide in 0.1 M Na-cacodylate (pH 7.4) for 1 hour. After washing with milli-Q H_2_O, pellets were incubated with 2% (w/v) uranylacetate for 1 hour. Fixed pellets were subsequently dehydrated in graded series of acetone (50–100%) and embedded in Durcupan ACM resin (Fluka, Bachs, Switzerland). Ultrathin sections of 50 nm were obtained on a Reichert UltracutS (Leica Aktiengesellschaft, Vienna, Austria) and studied using TEM (Philips CM 10, Philips, Eindhoven, the Netherlands).

### Biochemical analysis

Alkaline phosphatase was measured in samples that were incubated in a buffer containing 200 mM glycine, 2 mM MgCl_2_ and 20 mM *p*-nitrophenylphosphate with 0.05% Triton X-100 (pH 9.9) for 15 minutes, 37°C. The reaction was stopped by adding 0.5 M NaOH and subsequently measured in a spectrophotometer (Benchmark™ micro plate reader, Bio-Rad Laboratories, Inc.) at 405 nm [46]. Acrosin activity was assayed by its esterolytic activity on N-α-benzoyl-*L*-arginine-*p*-nitroanalide hydrochloride (*L*-BAPNA, 4 mM, Sigma, St. Louis, MO) as described by Breden et al [47]. Samples were incubated in a buffer containing 55 mM Hepes, 55 mM NaCl and 0.01% (v/v) Triton X-100 (pH 8.0) for 2 hours, 22°C. The reaction was stopped by 500 mM benzamidine. The formation product of *p*-nitroanalide was measured continuously at 410 nm during the incubation in a buffer containing 0.1 M Tris (tris[hydroxymethyl]aminomethane) and 67 mM NaCl (pH 8.0) at 25°C.

### Quantification of lectin binding

An enzyme linked lectin binding assay (ELLBA) was performed according to Flesch et al. [19] to quantify lectin binding of the samples. ELISA plates were coated with 500 ng of protein in 0.1 M Na_2_CO_3_ (pH 9.6) overnight at 4°C. Plates were blocked for 10 minutes with 0.3% (v/v) Tween-20 in HBS and subsequently blocked for another hour in 0.05% (v/v) Tween-20 and 1% (v/v) casein in HBS. Biotin-PNA (0.5 µg/ml) was used to quantify the amount of OAM material in cavitated membrane fractions. Horseradish peroxidase conjugated streptavidin (0.1 µg/ml) was allowed to bind to the Biotin-PNA conjugates. 3,3′,5,5′ - tetramethylbenzidine was used as the substrate for HRP and bound enzyme activity was measured in a ELISA plate reader at 450 nm with 655 nm for reference filter [48]. Calculations were made with OD_450_ corrected for background absorption (sample without Biotin-PNA conjugate).

### Immuno-blotting

Protein concentration in boar sperm and boar brain samples was determined and standardized for all experiments according to the Lowry method [49]. For both sperm and control brain samples, equal amount of total protein extract was re-suspended with appropriate amount of lithium dodecyl sulfate (LDS) loading buffer (Invitrogen, Carlsbad, CA, USA). Samples were separated into (1) non-reducing condition: in the absence of reducing agent and without boiling treatment; (2) reducing conditions: in the presence of 0.1 M dithiothreitol (DTT) and heated for either 5 minutes at 90°C or 10 minutes at 100°C prior to Western blotting. Proteins were separated in a 4% stacking and 12% running SDS (sodium dodecyl sulfate)-PAGE gel and wet-blotted onto polyvinylidene difluoride (PVDF) membranes (GE Healthcare). After blocking for 1 hour with ReliaBLOT® at RT, blots were incubated with primary antibodies diluted in ReliaBLOT® for overnight at 4°C. After washing the blots in TBS (Tris buffered solution) with 0.2% v/v Tween-20 (TBST), secondary antibodies were subsequently added for 60 minutes. After rinse with TBST, protein staining was visualized by using chemiluminescence (ECL-detection kit; Supersignal West Pico, Pierce, Rockford IL, USA).

### Co-immunoprecipitation (IP)

Cavitated membrane fractions from capacitated and control sperm cells were subjected to strong solubilisation: samples were first lysed with 1% (w/v) SDS solution in HBS for 30 minutes, then lysed with 5% (v/v) Nonidet-P40 (NP-40) in HBS for another 30 minutes. Solubilised membrane suspensions were subsequently centrifuged (10000 g, 20 minutes). Supernatants were collected and measured for protein concentration prior to immunoprecipitation. For IP procedures, lysed membrane supernatants were first subjected to pre-clearing matrix (Santa Cruz Biotechnology) for 30 minutes at 4°C. To form the antibody-IP matrix complex, 100 µl of 25% (v/v) IP matrix suspension (Santa Cruz Biotechnology) was pre-incubated with 10 µg of antibody on an end-to-end rotor overnight at 4°C. Antibody-coated beads were rinsed with phosphate buffered saline (PBS, pH 7.4) to remove unbound antibody, pre-cleared membrane suspensions were then added to the antibody-IP matrix mixture and rotated overnight at 4°C. After IP, the complex was pelleted by centrifugation (200 g, 5 minutes); the supernatants were used to check the efficiency of the IP reaction. The pellet was subsequently rinsed with PBS. Appropriate amounts of freshly prepared LDS loading buffer was added after the last wash. To improve the detection of immunoprecipitated proteins assayed via Western blotting, IP/Western Blot reagents (ReliaBLOT®) were used to reduce the recognition of the IgG heavy/light chain at 55 and 25 kDa, respectively.

### Statistical analysis

Statistical analyses were carried out in SPSS 12.0. Statistical significance for 2-tailed tests is set at *p*≤0.05. For two-group comparison, non-parametric test for two-independent-samples (Mann-Whitney *U*-test) was used.

## Supporting Information

Table S1 (0.06 MB DOC)

Figure S1 Control and *in vitro* capacitation treatments did not result in spontaneous acrosome reactions. Percoll washed sperm suspensions were diluted in control (upper right) or capacitation (lower right) media and stained for cell integrity (propidium iodide on the Y-axis) and for spontaneous acrosome reaction (PNA-FITC X axis) as described before [20], [44]. The percentage of acrosome intact and life sperm are indicated ± SD (n = 4) and were not significantly different.
(0.45 MB TIF)

Figure S2 Stability properties of the 80 kDa trans-SNARE complex formed in capacitated sperm cells. Cavitated apical membranes (285000 g pellet) from capacitated sperm cells were treated with either 0.1 M DTT at 37°C or without this agent at 90 or 100°C. The 80 kDa band containing SNAP23, syntaxin 1B and VAMP3 (not shown) was insensitive for these treatments. The combination of 0.1 M DTT and 100°C resulted in full dissociation (not depicted here but see Figs 4–7).
(3.00 MB TIF)

Figure S3 Capacitation-independent involvement of VAMP 1 and VAMP 2 in the SNARE protein complex. Apical membranes of control and *in vitro* capacitated sperms were isolated via nitrogen cavitation in combination with differential ultracentrifugation and used for the detection of other two VAMP isoforms. All samples were loaded under either non-reducing condition or treated with reducing agent (0.1 M DTT) and heated at 90°C for 5 minutes. Both VAMP 1 and VAMP 2 appeared in 80 kDa SDS-resistant protein complexes under non-reducing condition irrespectively to the capacitation treatment. These protein complexes partially dissociated into an intermediate 45–55 kDa VAMP1/2-containing protein bands under 90°C heat-treated condition with 0.1 M DTT. 10 µg of total protein extract was used for all samples.
(0.58 MB TIF)
