# Supplementary material for: How Pig Sperm Prepares to Fertilize: Stable Acrosome Docking to the Plasma Membrane
Source: PLoS One. 2010 Jun 18;5(6):e11204. doi: 10.1371/journal.pone.0011204 (PMC2887851; doi:10.1371/journal.pone.0011204)
Supplement: Table S1 — (0.06 MB DOC) [file pone.0011204.s001.doc]

**Table S1**

(A) Quantification analysis on TEM morphology in correspondence to figure 3A

| **Section number** | | **1** | **2** | **3** | **SD** | **# sperm** |
| --- | --- | --- | --- | --- | --- | --- |
| **Control** | **loose structure** | **27** | **32** | **31** | **2.7** | **90** |
|  | **tight arrangement** | **4** | **3** | **3** | **0.6** | **10** |
|  | **total count** | **31** | **35** | **34** |  | **100** |
| **Capacitation** | **loose structure** | **9** | **7** | **11** | **2** | **27** |
|  | **tight arrangement** | **25** | **23** | **25** | **1.2** | **73** |
|  | **total count** | **34** | **30** | **36** |  | **100** |

(B) Quantification analysis on TEM morphology in correspondence to figure 3B

| **Section number** | | **1** | **2** | **3** | **SD** | **# sperm** |
| --- | --- | --- | --- | --- | --- | --- |
| **Control** | **OAM intact** | **28** | **31** | **26** | **2.5** | **85** |
|  | **OAM lost** | **4** | **7** | **4** | **1.7** | **15** |
|  | **total count** | **32** | **38** | **30** |  | **100** |
| **Capacitation** | **OAM intact** | **2** | **2** | **1** | **0.6** | **5** |
|  | **OAM lost** | **32** | **35** | **28** | **3.5** | **95** |
|  | **total count** | **34** | **37** | **29** |  | **100** |

(C) Quantification analysis on TEM morphology in correspondence to figure 3C

| **Section number** | | **1** | **2** | **3** | **SD** | **# vesicles** |
| --- | --- | --- | --- | --- | --- | --- |
| **Control** | **unilamellar** | **29** | **24** | **27** | **2.5** | **80** |
|  | **bilamellar** | **7** | **8** | **5** | **1.5** | **20** |
|  | **total count** | **36** | **32** | **32** |  | **100** |
| **Capacitation** | **unilamellar** | **4** | **4** | **3** | **0.6** | **11** |
|  | **bilamellar** | **31** | **29** | **29** | **1.2** | **89** |
|  | **total count** | **35** | **33** | **32** |  | **100** |
