# Supplementary figures and images for: How Pig Sperm Prepares to Fertilize: Stable Acrosome Docking to the Plasma Membrane
Source: PLoS One. 2010 Jun 18;5(6):e11204. doi: 10.1371/journal.pone.0011204 (PMC2887851; doi:10.1371/journal.pone.0011204)

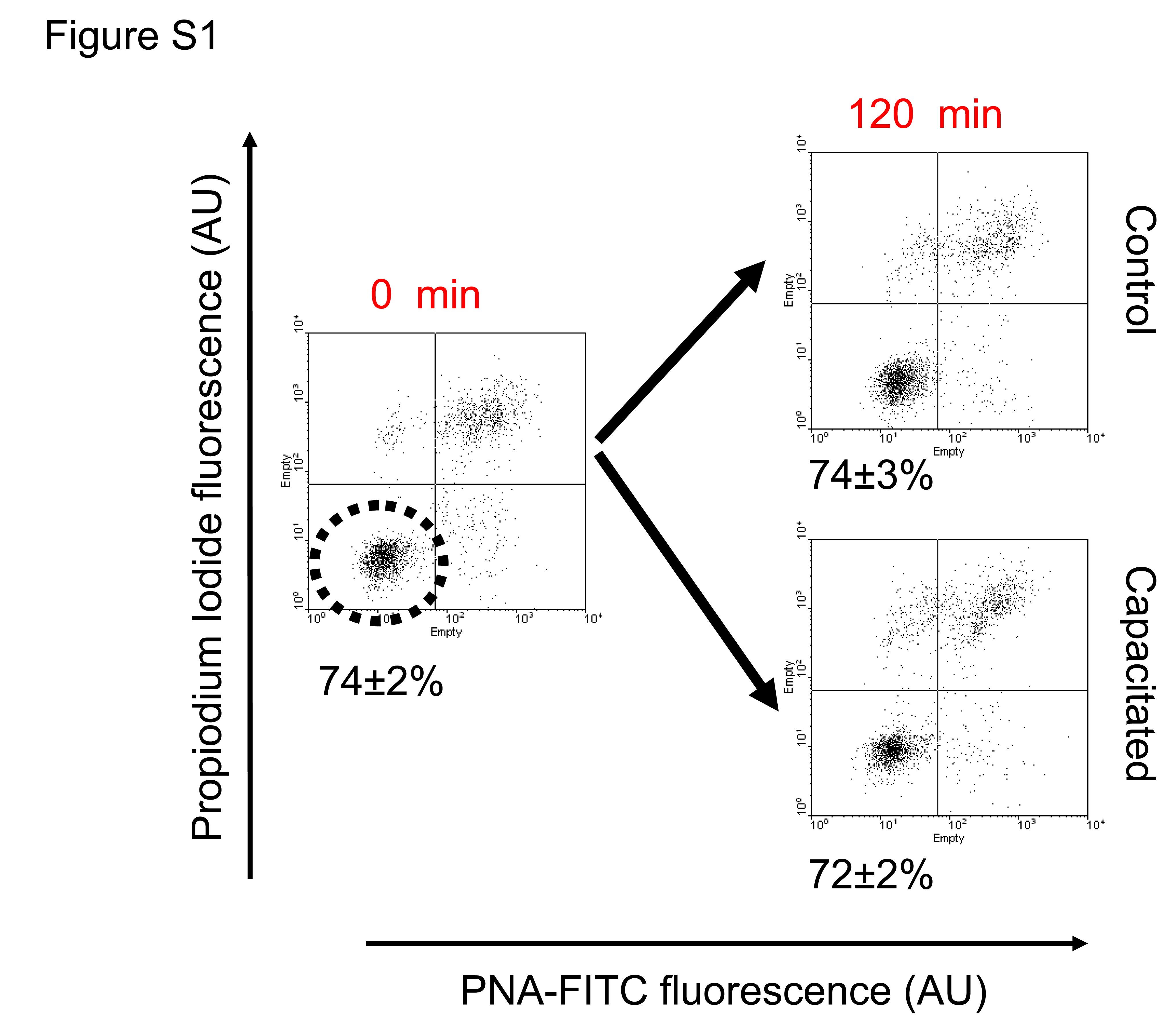

Supplement: Figure S1 — Control and in vitro capacitation treatments did not result in spontaneous acrosome reactions. Percoll washed sperm suspensions were diluted in control (upper right) or capacitation (lower right) media and stained for cell integrity (propidium iodide on the Y-axis) and for spontaneous acrosome reaction (PNA-FITC X axis) as described before [20], [44]. The percentage of acrosome intact and life sperm are indicated ± SD (n = 4) and were not significantly different. (0.45 MB TIF) [file pone.0011204.s002.tif]

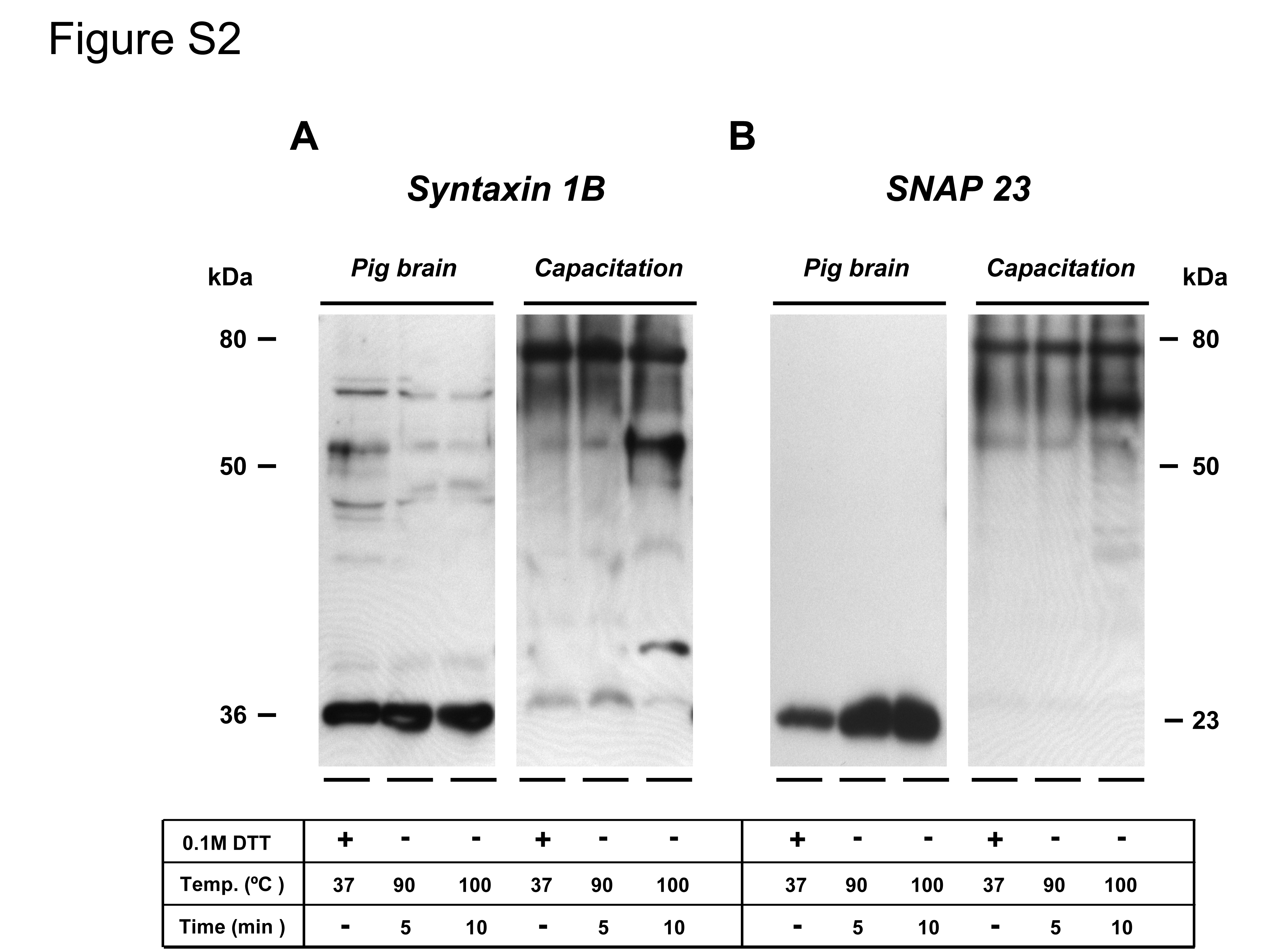

Supplement: Figure S2 — Stability properties of the 80 kDa trans-SNARE complex formed in capacitated sperm cells. Cavitated apical membranes (285000 g pellet) from capacitated sperm cells were treated with either 0.1 M DTT at 37°C or without this agent at 90 or 100°C. The 80 kDa band containing SNAP23, syntaxin 1B and VAMP3 (not shown) was insensitive for these treatments. The combination of 0.1 M DTT and 100°C resulted in full dissociation (not depicted here but see Figs 4– 7). (3.00 MB TIF) [file pone.0011204.s003.tif]

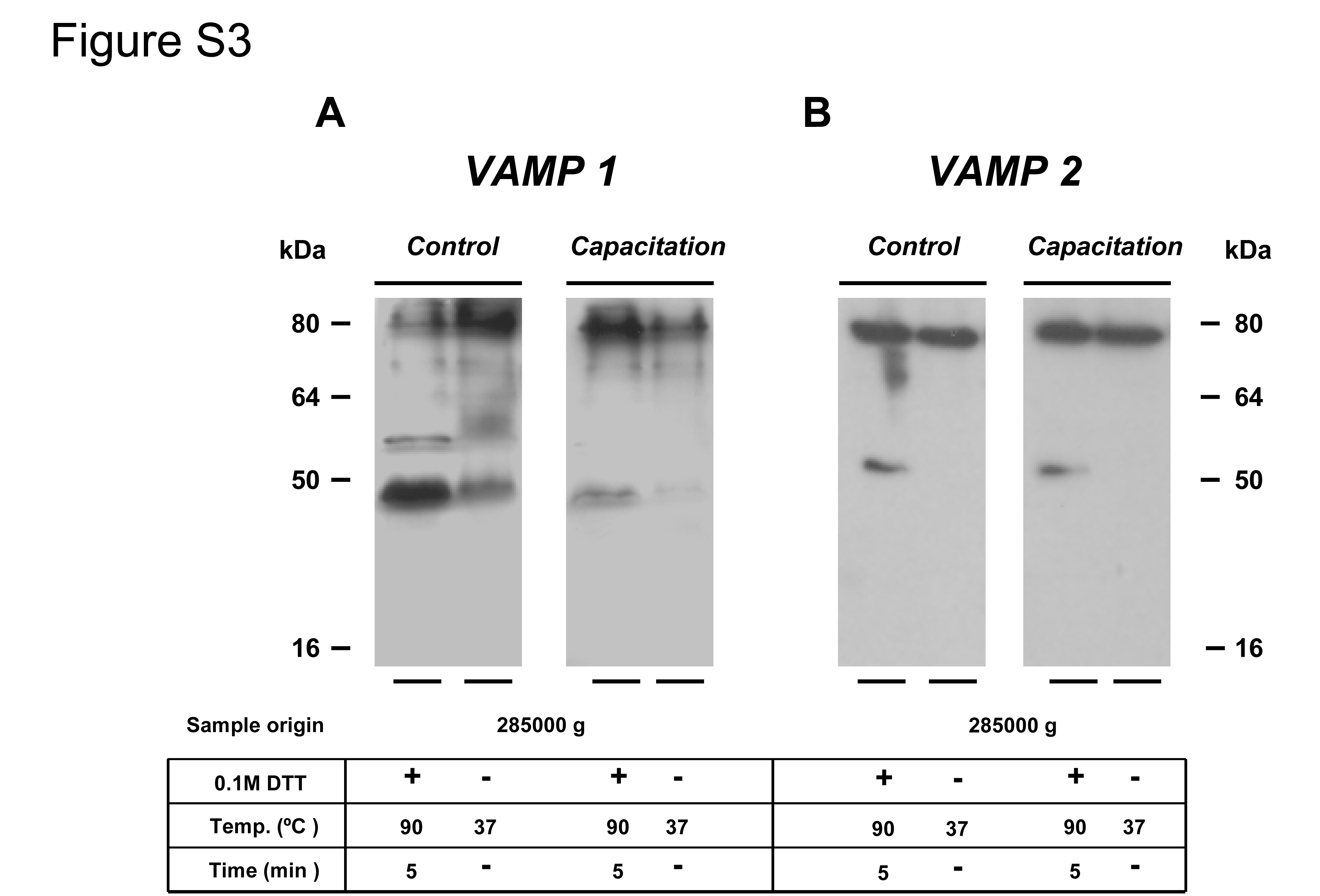

Supplement: Figure S3 — Capacitation-independent involvement of VAMP 1 and VAMP 2 in the SNARE protein complex. Apical membranes of control and in vitro capacitated sperms were isolated via nitrogen cavitation in combination with differential ultracentrifugation and used for the detection of other two VAMP isoforms. All samples were loaded under either non-reducing condition or treated with reducing agent (0.1 M DTT) and heated at 90°C for 5 minutes. Both VAMP 1 and VAMP 2 appeared in 80 kDa SDS-resistant protein complexes under non-reducing condition irrespectively to the capacitation treatment. These protein complexes partially dissociated into an intermediate 45–55 kDa VAMP1/2-containing protein bands under 90°C heat-treated condition with 0.1 M DTT. 10 µg of total protein extract was used for all samples. (0.58 MB TIF) [file pone.0011204.s004.tif]
